# Morphological and Cellular Features of Innate Immune Reaction in *Helicobacter pylori* Gastritis: A Brief Review

**DOI:** 10.3390/ijms17010109

**Published:** 2016-01-15

**Authors:** Antonio Ieni, Valeria Barresi, Luciana Rigoli, Francesco Fedele, Giovanni Tuccari, Rosario Alberto Caruso

**Affiliations:** 1 Department of Human Pathology “*Gaetano Barresi*”, University of Messina, Messina 98125, Italy; 2 Azienda Ospedaliera Universitaria “*Policlinico Gaetano Martino*”, Messina 98125, Italy; aieni@unime.it (A.I.); lrigoli@unime.it (L.R.); f.fedele1965@libero.it (F.F.); tuccari@unime.it (G.T.); rosariocaruso@tin.it (R.A.C.)

**Keywords:** innate immunity, gastritis, *Helicobacter pylori*, neutrophils, mast cell, eosinophils

## Abstract

Innate and adaptive immunity are both involved in acute and chronic inflammatory processes. The main cellular players in the innate immune system are macrophages, mast cells, dendritic cells, neutrophils, eosinophils, and natural killer (NK), which offer antigen-independent defense against infection. *Helicobacter pylori* (*H. pylori*) infection presents peculiar characteristics in gastric mucosa infrequently occurring in other organs; its gastric colonization determines a causal role in both gastric carcinomas and mucosa-associated lymphoid tissue lymphoma. In contrast, an active role for Epstein-Barr virus (EBV) has been identified only in 9% of gastric carcinomas. The aim of the present review is to discuss the role of cellular morphological effectors in innate immunity during *H. pylori* infection and gastric carcinogenesis.

## 1. Introduction

The immune system is based on innate and adaptive immunity, both participating in acute and chronic inflammatory reactions. An inflammatory response occurs via antigen-dependent or -independent stimulation. Antigen-dependent pathways involve adaptive immunity that is represented by T and B lymphocytes, clonally expressing antigen receptors that are produced by site-specific somatic recombination, *i.e.*, T cell receptor and antibody/B cell receptor [1]. Macrophages, mast cells, dendritic cells, neutrophils, eosinophils, and natural killer (NK) cells are cell actors of the innate immune system that provides antigen-independent defense against infection [2].

Epidemiological and experimental data showed that chronic inflammation predisposes patients to different types of cancer [3]. The etiology of chronic inflammation can be infective, such as virus, bacteria, or parasites or may be non-infective irritant, either physical and chemical [3]. The percentage of total cancer deaths due to infectious agents ranges from 7% to 10% in Western countries, while its value is about 20%–25% in developing countries [3]. Virus-associated cancers include hepatocellular carcinomas induced by hepatitis C virus and hepatitis B virus, cervical cancers induced by human papilloma virus, and lymphomas and nasopharyngeal cancers associated with Epstein-Barr virus (EBV) [3].

Gastric carcinoma is a paradigmatic example of infection-associated cancer because the majority of cases are due to *Helicobacter pylori* (*H. pylori*) and EBV [4,5]; however, the incidence of *H. Pylori* and EBV associated gastric cancer is not homogeneous worldwide [5]. EBV has been identified within malignant epithelial cells in 9% of gastric carcinomas, and EBV-associated gastric cancer has been defined as one major genomic subtype of gastric cancer, together with microsatellite instability (MSI), genomically stable and chormosomomally unstable subtypes [5]. Nevertheless, a causal role in both gastric carcinomas and mucosa-associated lymphoid tissue lymphoma has been assigned to the colonization with *H. pylori* [4]. In this field, the morpho-histopathological identification of bacteria as well as cellular inflammatory actors may provide a guide to targeted agents, that should determine the *H. Pylori* eradication, with a decreased incidence of gastric cancer.

This review focuses on the main morphological and cellular findings concerning the involvement of innate immunity in both *H. pylori* infection and gastric carcinogenesis.

## 2. Innate Immune Cells in *H. pylori* Gastritis

Inflammatory reaction to *H. pylori* infection shows “*sui generis*” characteristics and occurs rarely in other organs or biological systems. Classically, outcomes of acute inflammation include resolution, abscess formation, fibrosis, or chronic inflammation. Instead, a mixed acute and chronic inflammatory reaction takes place at the same time during *H. pylori* gastritis (Table 1), where both neutrophils and lymphocytes, as well as macrophages and plasma cells infiltrate the mucosa in a characteristic manner [6,7,8]. In fact, neutrophils [4], mast cells [6,7], eosinophils [4], and dendritic cells [8] may directly infiltrate foveolar epithelium, whereas the lamina propria is permeated by mononuclear cells, such as lymphocytes, macrophages and plasma cells [4].

**Table 1:** Cellular players of innate immunity in *H. pylori* gastritis.

Cellular Actors	Role
Neutrophils	Marker of active disease
Mast cells	Starter of acute inflammatory reaction
Eosinophils	Producer of pro-fibrogenic/angiogenic factors
Macrophages	Scavenger of pathogens
Dendritic cells	Promoter of chronic infection

## 3. Role of Neutrophils in *H. pylori* Gastritis

**Figure 1:**
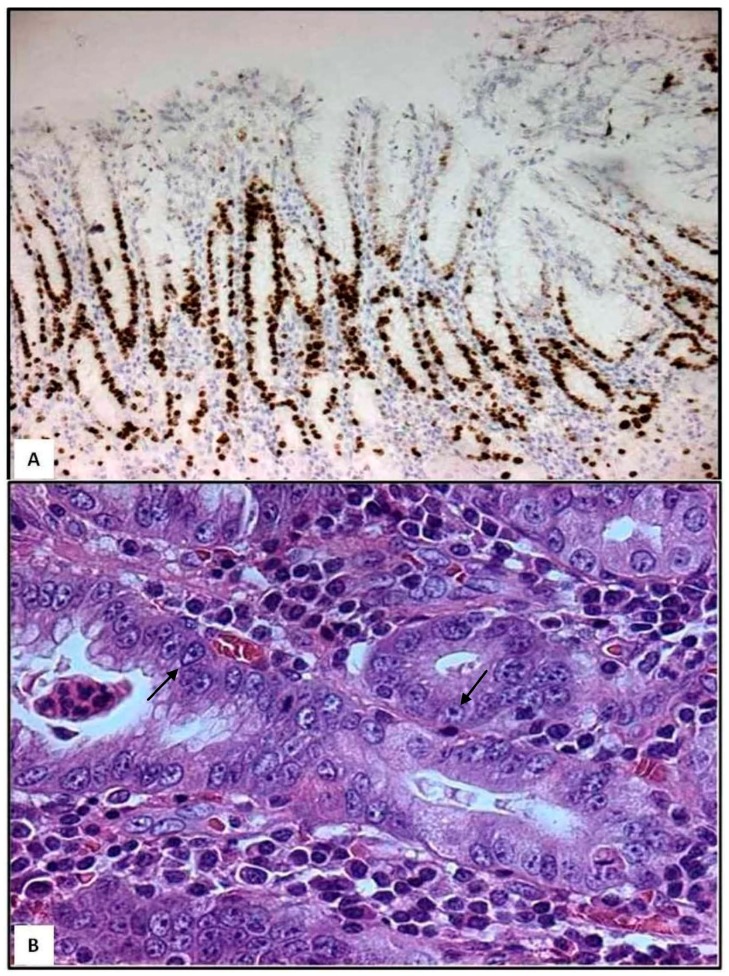
Gastric bioptic specimens—Ki-67-immunostained cycling epithelial cells in the deep foveolar zone and in the isthmus/neck region; few Ki-67-positive cells are present in deep glandular zone (**A**, immunoperoxidase, Mayer’s haemalum counterstain, 160×). Epithelial cells of the proliferative zone exhibit enlarged nuclei containing prominent nucleoli (arrows) and cytoplasmic mucin loss (**B**, haematoxylin and eosin, 400×). (Unpublished personal data).

Following the progression of *H. pylori* gastritis, gastric mucosa acquires irregular morphology due to crowding of pits [9]. Moreover, by Ki-67 immunohistochemistry, an increased proliferation is found in the gastric mucosa of bioptic specimens [9]. In detail, cycling epithelial cells are found at the isthmus/neck region and at the deep portion of the pits (Figure 1A); this proliferative zone appears elongated and constituted by epithelial cells showing enlarged nuclei, prominent nucleoli, and loss of cytoplasmic mucins (Figure 1B). From a histopathological viewpoint, presence of neutrophils characterizes the activity and severity of *H. pylori* gastritis [4]. In parallel, neutrophils infiltrate selectively the aforementioned proliferative zones and spare surface and deep glandular zones [9]. However, in some cases, neutrophils may infiltrate surface zones, where they may form pit abscesses [9]. During trans-epithelial migration, some neutrophils undergo apoptosis and are phagocytosed by foveolar cells [10]; this migration of neutrophils is also associated with sub-lethal or lethal injury of foveolar cells [9,10]. If neutrophils damage large segments of foveolar epithelium, then mucosal erosion occurs [9], as elsewhere suggested by the surface neutrophilic foveolitis, usually happening in patients with recurrent erosions and/or ulcers [9].

In contrast with classic acute inflammation, where neutrophil infiltration is self-limited, chronic neutrophilic infiltration targeting the proliferative zone has strong patho-biological implications [9].

It has been hypothesized that infiltration of neutrophils may regard the proliferative zone where the epithelial junctions are not so strong in comparison to surface or deep glandular areas [9] and therefore *H. pylori* is able to quickly modify the epithelial normal structure.

Moreover, intraepithelial neutrophils generate reactive oxygen and nitrogen species inducing genomic changes in vulnerable cycling or mitotically active epithelial cells of the proliferative zone [9].

## 4. Role of Mast Cells in *H. pylori* Gastritis

Generally speaking, mast cells play an important role in acute inflammatory processes, allergic hypersensitivity type I reaction, tissue remodeling, wound healing and angiogenesis [11]. Mast cells occupy strategic positions throughout the body, being present in areas exposed to the external environment, such as skin, mucosa of the gastrointestinal, respiratory, and genitourinary tracts, where they interact with invading pathogens [11]. Moreover, mast cells are located near vessels in the connective tissue where they may easily recruit circulating leukocytes [12]. Secretory granules of human mast cells store biogenic amines (histamine and serotonin), preformed cytokines (tumor necrosis factor-α), serglycin proteoglycans, various lysosomal enzymes and some specific proteases, such as chymase, tryptase and carboxypeptidase A [11]. The release of histamine from mast cells induces vasodilatation and edema, whereas the release of tumor necrosis factor-α, prostaglandin D2 and interleukin-8 stimulates neutrophil chemotaxis [12]. Therefore, mast cell mediators play an important role in initiating the acute inflammatory reaction.

**Figure 2:**
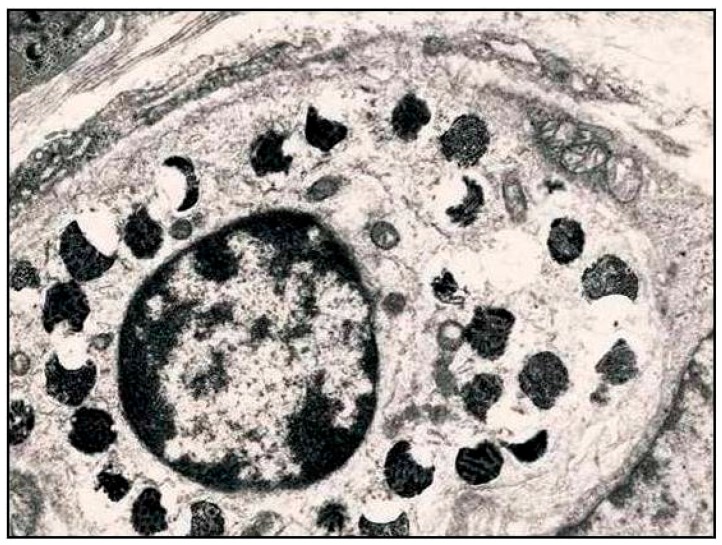
Intraepithelial mast cell showing partially empty, slightly enlarged, non-fused granules containers, a process similar to piecemeal degranulation; this latter phenomenon may represent a picture related to acute inflammatory reaction during *H. pylori* infection. (TEM, 8000×—Unpublished personal data).

Rarely, mast cells are able to infiltrate the epithelia in normal gastric mucosa, although intraepithelial infiltration increases in certain inflammatory states including *H. pylori* gastritis [6,7]. Particularly, by ultrastructural observations, we have revealed that mast cells may actively migrate into the gastric epithelium, where they undergo degranulation (Figure 2) [7]. This intraepithelial strategic location, usually overlooked in current literature, may be useful to evoke an acute inflammatory reaction more easily during *H. pylori* infection [7]. Furthermore, it has been shown that the number of mast cells was increased in patients with *H. pylori* gastritis and it was dependent on disease activity [13]. In addition, the density of mast cells has been documented as higher in patients infected with aggressive *H. pylori* strains (cagA, vacAs1/mL, babA2, and triple-positive *H. pylori* strains) as compared to controls, suggesting the opportunity to consider the density of mast cells as a further morphologic evidence of activity in *H. pylori* gastritis [13].

## 5. Role of Eosinophils in *H. pylori* Gastritis

Human eosinophils are multifunctional granulocytes involved in allergy, helminth infection and host-tumor reaction. Although high densities of eosinophils are found in chronic active *H. pylori* gastritis and after eradication therapy for *H. pylori*, the updated Sydney system did not recommend to pathologists routine grading of eosinophils in *H. pylori* gastritis [4]. However, a redistribution of eosinophils in *H. pylori* gastritis has been documented [14]; in particular, an increased density of eosinophils has been reported in the superficial layer of the lamina propria [14]. Intraepithelial eosinophil infiltration may also occur and this finding must always be interpreted as abnormal according to the updated Sydney system [4]. On the other hand, in areas of gastric atrophy, an increased density of both eosinophils and mast cells has been documented [15]; these inflammatory elements appears to play a significant role in the pathogenesis of gastric atrophy due to the release of pro-fibrogenic and pro-angiogenic factors, leading thus to tissue remodeling and fibrosis [15].

## 6. Role of Macrophages in *H. pylori* Gastritis

Traditionally, *H. pylori* was considered to be a non-invasive pathogen that resides on gastric epithelial cell surface and in the overlying mucus*.* Recent studies revealed that *H. pylori* is invasive and shows facultative intracellular bacterial behavior. In particular, *H. pylori* invades not only epithelial cells, but also macrophages, neutrophils, and dendritic cells [16,17]. It is well known that engulfment of particulate material and microorganisms is defined as phagocytosis, a phylogenetically conserved process [18]. On the other hand, pathogen clearance results from several sequential processes, including detection and binding of the pathogen, phagocytosis, phagosome maturation, lysosomes targeting and ultimate destruction [18]. Traditionally, autophagy was considered a nonselective mechanism in which cytoplasmic organelles are engulfed and degraded by lysosomes, playing an important role in the defense against bacterial invasion [19]. In addition to the clearance of damaged organelles and protein aggregates, autophagosomes may also eliminate intracellular microbes [20]. Moreover, it has been demonstrated that *H. pylori* was able to manipulate autophagic mechanisms to replicate and survive within host cells [21]; in particular, virulence factors of *H. pylori* play a key role in the prolonged bacterial survival within macrophages [21]. In contrast, avirulent type 2 CagA-negative, VacA-negative *H. pylori* strains undergo a fully degradative process within the phagolysosome compartment [22]. Virulent type 1 strains (VacA-positive, CagA-positive) of *H. pylori* instead arrest the maturation of phagosome, generating large autophagosomes (also defined megasomes) [22]. Finally, macrophages infected with *H. pylori* undergo apoptosis with subsequent bacterial liberation [23,24]; consequently, the induction of macrophage apoptosis represents a mechanism employed by virulent strains of *H. pylori* to escape host immune response and favor bacterial persistence [24].

## 7. Role of Dendritic Cells in *H. pylori* Gastritis

Dendritic cell maturation is fundamental for antigen presentation and stimulation of T cells. Immature dendritic cells have tolerogenic properties and may faciitate chronic infection [25]. Ultrastructural and cytochemical studies have shown intraepithelial location of dendritic cells in *H. pylori* gastritis [8]. Intraepithelial dendritic cells contained in the cytoplasm bacterial products such as VacA and urease [8]. Several intraepithelial dendritic cellsl showed ultrastructural signs of sublethal or lethal injury, suggested by the presence of focal mitochondrial swelling, cytoplasmic edema, vacuolization and autophagic vacuoles [8]. These morphologic findings, corroborated by *in vitro* studies on dendritic cells incubated with *H. pylori*, are compatible with an impairment of dendritic cell function due to accumulated bacterial toxins [26]. Therefore, they may explain the impaired or suboptimal dendritic cell response with consequent persistence of *H. pylori* infection.

## 8. Conclusions

Recent advances have modified traditional views about the involvement of innate immunity in *H. pylori*-associated gastric carcinogenesis. Salient findings presented in this review are as follows:

- Histopathological and ultrastructural studies have revealed that not only neutrophils and eosinophils, but also mast cells and dendritic cells may directly infiltrate gastric foveolar epithelium during *H. pylori* infection. Therefore, these innate immune cells occupy strategic positions in order to evoke chronic active inflammation more easily.
- Although *H. pylori* was initially considered as a non-invasive pathogen, several studies have demonstrated that *H. pylori* is a facultative intracellular bacterium within epithelial cells, neutrophils, macrophages and dendritic cells.
- Highly virulent *H. pylori* strains arrest the normal phagosome maturation process in macrophages and dendritic cells, and generate large autophagosomes (also defined megasomes) where *H. pylori* can multiply, impairing immunological defense.

Finally, apoptosis or functional exhaustion of macrophages and dendritic cells can occur, with consequent persistence of *H. pylori* infection. Further studies on the role of innate immunity will be useful not only to gain a better understanding of the pathology of *H. pylori* infection but also to identify alternatives to antibiotic-based therapies for control of *H. pylori* gastritis and prevention of gastric neoplasms.
